# DNA methylation profiling at base-pair resolution reveals unique epigenetic features of early-onset colorectal cancer in underrepresented populations

**DOI:** 10.1186/s13148-025-01817-z

**Published:** 2025-01-22

**Authors:** Jason Sheng Li, Karen Riggins, Li Yang, Chaorong Chen, Patricia Castro, Wedad Alfarkh, Neda Zarrin-Khameh, Michael E. Scheurer, Chad J. Creighton, Benjamin Musher, Wei Li, Lanlan Shen

**Affiliations:** 1 Division of Computational Biomedicine, Department of Biological Chemistry, School of Medicine, University of California, Irvine, CA 92697 USA; 2 Department of Medicine, Hematology and Oncology, Dan L. Duncan Comprehensive Cancer Center, Baylor College of Medicine, Houston, TX 77030 USA; 3 Department of Pediatrics, USDA Children’s Nutrition Research Center, Baylor College of Medicine, Houston, TX 77030 USA; 4 Department of Pathology and Immunology, Baylor College of Medicine, Houston, TX 77030 USA; 5 Department of Pathology, Ben Taub Hospital, 1504 Taub Loop, Houston, TX 77030 USA; 6 Department of Pediatrics, Center for Epidemiology and Population Health, Baylor College of Medicine, Houston, TX 77030 USA; 7 Department of Medicine and Dan L Duncan Comprehensive Cancer Center, Baylor College of Medicine, Houston, TX 77030 USA; 8 Department of Medicine, Gastrointestinal Medical Oncology, Dan L. Duncan Comprehensive Cancer Center, Baylor College of Medicine, Houston, TX 77030 USA

**Keywords:** Cancer epigenetics, Early-onset colorectal cancer, DNA methylation, Cancer disparity

## Abstract

**Background:**

The incidence of early-onset colorectal cancer (EOCRC) has been rising at an alarming rate in the USA, and EOCRC disproportionately affects racial/ethnic minorities. Here, we construct comprehensive profiles of EOCRC DNA methylomes at base-pair resolution for a cohort of Hispanic and African American patients.

**Results:**

We show the epigenetic landscape of these EOCRC patients differs from that of late-onset colorectal cancer patients, and methylation canyons in EOCRC tumor tissue preferentially overlapped genes in cancer-related pathways. Furthermore, we identify epigenetic alterations in metabolic genes that are specific to our racial/ethnic minority EOCRC cohort but not Caucasian patients from TCGA. Top genes differentially methylated between these cohorts included the obesity-protective *MFAP2* gene as well as cancer risk susceptibility genes *APOL3* and *RNASEL*.

**Conclusions:**

In this study, we provide to the scientific community high-resolution DNA methylomes for a cohort of EOCRC patients from underrepresented populations. Our exploratory findings in this cohort highlight epigenetic mechanisms underlying the pathogenesis of EOCRC and nominate novel biomarkers for EOCRC in underrepresented populations.

**Supplementary Information:**

The online version contains supplementary material available at 10.1186/s13148-025-01817-z.

## Background

While colorectal cancer (CRC) typically presents after the age of 50 (late-onset colorectal cancer [LOCRC]), epidemiologic data have shown a disturbing rise in the incidence of colorectal cancer among individuals younger than 50 (early-onset colorectal cancer [EOCRC]) [1]. In fact, current trends indicate that, over the next decade, EOCRC will account for 25% of rectal cancers and 10–12% of colon cancers, making it the leading cause of cancer-related mortality in persons aged 20–49 years [2]. Despite the rising incidence of EOCRC, clear clinico-histopathological differences, and the recognition that EOCRC likely represents a distinct disease with a unique molecular landscape and oncogenic mechanisms, no clear etiology of this emerging disease has been identified [3].

Approximately 80% of EOCRCs are sporadic, with no underlying hereditary conditions such as Lynch syndrome [4]. Compared to LOCRC, EOCRC arises predominantly on the left side of the colon and is associated with more aggressive histology and advanced stage at diagnosis [5]. At the molecular level, EOCRC patients are more likely to have somatic mutations in *TP53* and *CTNNB1*, whereas *APC* and *BRAF* are less frequently mutated [6]. In addition, recent transcriptomic and metabolomic profiling has implicated glutathione metabolism and NRF2-mediated oxidative stress in the pathogenesis of microsatellite-stable EOCRC, underscoring the need for unbiased methods to characterize molecular signatures differentiating EOCRC from LOCRC [7].

In the USA, racial and ethnic minorities are disproportionately affected by EOCRC [8]. For example, compared to Caucasians, the incidence of EOCRC has increased at a faster rate among Hispanics and African Americans with significantly lower 5-year survival [9]. The causes of these disparities are unclear, but environmental risk factors such as diet, stress, and gut microbiome likely contribute to the differences in incidence and mortality among races [10, 11]. One potential mechanism is through environmental influences on epigenetic modifications that affect gene expression without changing the underlying DNA sequence. Unfortunately, this remains understudied in translational research.

DNA methylation, which results from the addition of methyl groups to cytosine-guanine dinucleotides (CpGs), is a key epigenetic mediator of expression. Dysregulation of DNA methylation plays an important role in the pathogenesis of CRC [12, 13]. Indeed, whole-genome DNA methylation profiling reveals that cancer cells acquire widespread changes in DNA methylation to upregulate oncogenic programs and repress tumor-protective programs [14]. Recently, the existence of methylation canyons, large (> 3.5 kb) undermethylated regions flanked by sharp peaks of high methylation, has been described [15]. Canyons are enriched in homeobox and Polycomb genes and are involved in transcriptional regulation in cancer, especially in oncogene activation [16, 17]. While DNA methylation microarrays, such as the Illumina Infinium BeadChips, have become increasingly popular because of their low cost and rapid profiling, they cover only a small fraction of all CpGs in the human genome and are unable to profile larger regions, including methylation canyons. On the other hand, whole-genome bisulfite sequencing (WGBS), which utilizes bisulfite conversion of methylated cytosines, offers unparalleled base-pair resolution of the entire methylome at a higher cost. A comprehensive WGBS analysis of EOCRC, however, has not yet been reported.

In addition to the relatively high cost of generating whole genome datasets, racial/ethnic minority patients are also severely underrepresented in research studies, resulting in a dearth of high-quality datasets from these populations. For example, over 80% of patients included in The Cancer Genome Atlas (TCGA) are of European descent, whereas only ~ 9% are African American [18]. Studies seeking a reason for the low representation of racial/ethnic minorities in biorepositories and research studies point to patient distrust, language barriers, or researcher bias [19–21]. Irrespective of the reason, the result is a distinct lack of tumor biospecimens from underrepresented populations, presenting a major barrier to our understanding of diseases with disparities between racial and ethnic groups, such as EOCRC.

To address both the lack of high-quality sequencing datasets and the pressing unmet need for molecular characterization of EOCRC, as well as to explore the molecular basis of EOCRC racial disparities, we identified a cohort of Hispanic and African American patients with sporadic left-sided EOCRC and performed WGBS for 9 tumors with 7 matched normal adjacent tissue (NAT) samples. We compared genome-wide differential methylation in this cohort to both a Caucasian EOCRC cohort and a LOCRC cohort from TCGA. By leveraging the power of WGBS, combined with our unique cohort, we contribute the first high-quality complete EOCRC methylomes from racial/ethnic minority patients as a resource to researchers. We further provide an unbiased description of epigenetic changes during EOCRC oncogenesis, define unique regulatory methylation canyons, characterize the epigenetic landscape of epithelial-to-mesenchymal transition (EMT) in this EOCRC cohort, and identify biologically relevant risk genes in racial/ethnic minority patients.

## Results

### Global DNA methylation patterns in EOCRC

**Fig. 1:**
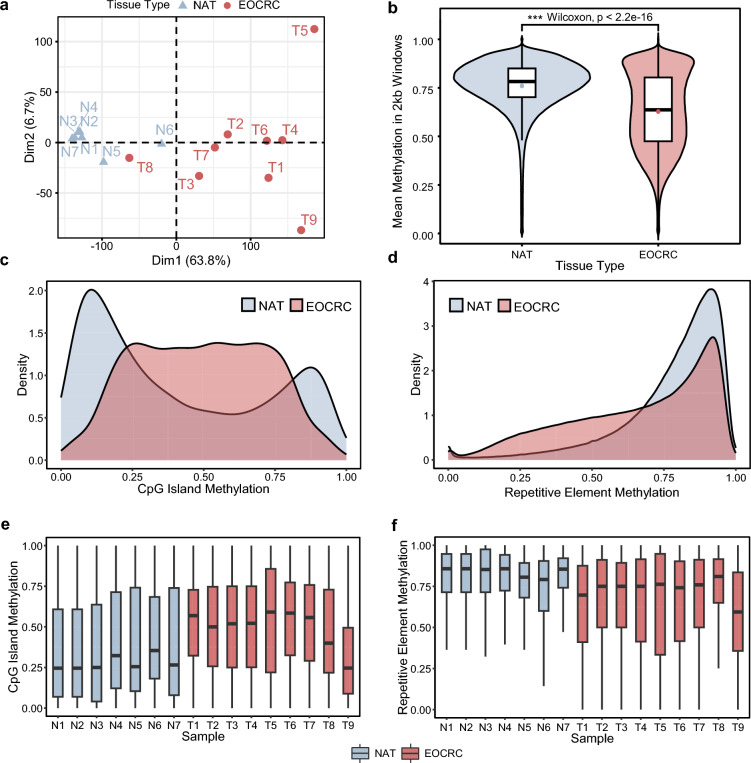
Global methylation characteristics of early-onset colorectal cancer. **a** Principal component analysis of methylation across 2-kb sliding windows for EOCRC tumor tissues (*n* = 9) and adjacent normal colonic mucosal tissues (*n* = 7). **b** Global methylation of aggregated samples across 2-kb sliding windows. EOCRC samples are globally hypomethylated, compared to NAT. **c** Density plot of CpG island methylation of aggregated samples showing extensive hypermethylation in EOCRC tissue. **d** Density plot of methylation at all repetitive elements in aggregated samples showing hypomethylation in EOCRC tissue. Boxplots of per-sample methylation at **e** CpG islands and **f** repetitive elements. *CpG* cytosine-guanine dinucleotide, *EOCRC* early-onset colorectal cancer, *N1 to N7* normal adjacent tissue 1 to 7, *NAT* normal adjacent tissue, *T1 to T9* tumor tissues 1 to 9

To first understand global methylation changes in EOCRC oncogenesis, we compared methylation between EOCRC tumors and matched NATs (Supplementary Table S1) across the entire genome by binning the genome into 2-kb sliding windows. EOCRC patients were selected using these inclusion criteria to exclude those arising from Lynch Syndrome or inflammatory bowel diseases (i.e., Crohn’s, ulcerative colitis): age of diagnosis < 50 years; mismatch repair (MMR)-proficient tumors; and nonhypermutated tumors (Supplementary Fig. S1). Based on the mean methylation of each sample at all 2-kb windows, we performed principal component analysis (PCA) and found that NAT clustered closely together, while EOCRC tumors were heterogeneous and distributed far away in PCA space from NAT, except for T8 (Fig. 1a). The clustering of sample T8 with NAT may reflect clinical confounders such as body mass index and comorbidities (e.g., type 2 diabetes), sample quality, and tumor heterogeneity. T8 was retained in subsequent analyses of the EOCRC cohort to more comprehensively reflect clinically relevant disease heterogeneity. Globally, tumor tissues were extensively hypomethylated (Fig. 1b), which is consistent with previous reports using a repetitive element LINE1 as a surrogate marker of global methylation in EOCRC [22–24]. In addition, the EOCRC tumors were highly hypermethylated at CpG islands compared to NAT (Fig. 1c), whereas repetitive elements (Alu, SINEs, LINEs) were overall hypomethylated (Fig. 1d). Similar trends were generally observed at a per-sample level, but with substantial heterogeneity in tumors (Fig. 1e and f).

### Differential methylation between EOCRC and normal adjacent tissue

We continued our unbiased comparison of EOCRC and NAT by performing differential methylation analysis using a circular binary segmentation algorithm from the tool Metilene [25]. To provide an overview of differential methylation, we performed both differentially methylated region (DMR) and differentially methylated CpG position (DMP) analysis in de novo mode. Regions differentially methylated between EOCRC and NAT groups were de novo annotated by circular binary segmentation and then filtered to keep only regions with ≥ 10 CpGs in the region and absolute methylation differences ≥ 0.1.

**Fig. 2:**
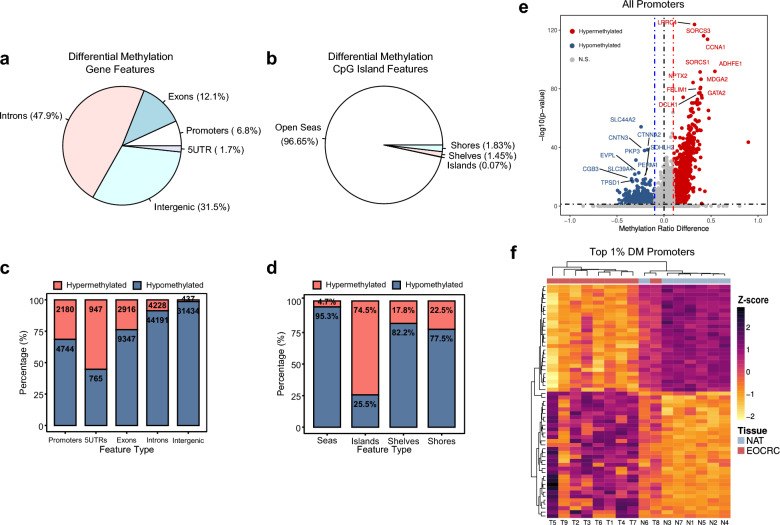
Landscape of differential methylation in early-onset colorectal cancer. **a** Pie chart of differentially methylated regions (DMRs) according to gene location. **b** Pie chart of differentially methylated CpGs (DMPs) according to CpG island features. **c** Proportions of DMRs hypermethylated and hypomethylated according to gene location. **d** Proportions of DMPs hypermethylated and hypomethylated according to CpG island features. **e** Volcano plot showing significance and methylation difference for all promoters. Labels indicate gene names for the top 10 most significant hypermethylated and hypomethylated promoters. **f** Heatmap of the top 1% most variable hypermethylated and hypomethylated promoter DMRs. Tile color indicates row-wise normalized Z-scores. Columns are annotated by tissue type. CpG, cytosine-guanine dinucleotide; DM, differentially methylated; EOCRC, early-onset colorectal cancer; N1 to N7, normal adjacent tissue 1 to 7; NAT, normal adjacent tissue; N.S., not significant; T1 to T9, tumor tissues 1 to 9; UTR, untranslated region

Filtered regions were evaluated using 2-dimensional Kolmogorov–Smirnov and Mann–Whitney U tests to yield a final DMR set with adjusted *p* value < 0.05. Most DMRs occurred in noncoding regions, including introns (47.9%) and intergenic regions (31.5%), but DMRs were also discovered in exons (12.1%) and promoters (6.8%) (Fig. 2a). Most noncoding region DMRs were hypomethylated, whereas promoters were more evenly split between hypomethylated and hypermethylated, favoring reduced methylation (Fig. 2c). These promoter methylation proportions resemble those previously observed in EOCRC using HM450K microarrays [26].

To study DMPs, we used the Mann–Whitney U test with Bonferroni correction to evaluate significant differences between EOCRC and NAT groups for individual CpGs with an absolute methylation difference ≥ 0.1. DMPs occurred predominately in open seas (96.65%), while the remaining DMPs occurred in CpG shores (1.83%), shelves (1.45%), and islands (0.07%) (Fig. 2b). The majority of intergenic DMPs were hypomethylated (95.3%), which in combination with the high proportion of DMPs in intergenic regions (including repetitive elements) likely accounted for most of the global DNA hypomethylation found in EOCRC (Fig. 2d).

Despite the canonical view of transcriptional repression by DNA methylation, the relationship between methylation and gene expression is complex and position dependent. DNA hypermethylation correlates with reduced gene expression mainly at promoters. This correlation is tenuous or even reversed in other gene regions, obfuscating biological interpretation from methylome data alone [27]. Thus, we focused primarily on promoters in subsequent DMR analyses, performing another differential methylation study between EOCRC and NAT restricted to promoter regions. To ensure equal comparison between samples, we defined promoter regions as a window from 1000 bp upstream to 500 bp downstream of the transcription start site (TSS). In total, 1,073 statistically significant promoter DMRs (pDMRs) were identified, 694 (64.7%) of which were hypermethylated while 379 (35.3%) were hypomethylated in EOCRC, compared to NAT (Fig. 2e). Unsupervised hierarchical clustering of samples by the top 1% most variable pDMRs generally separated tumors from NAT, although the previous PCA outlier T8 clustered with the NAT group (Fig. 2f).

### Methylation canyons overlap oncogenic pathway genes and are altered in colorectal cancer

**Fig. 3:**
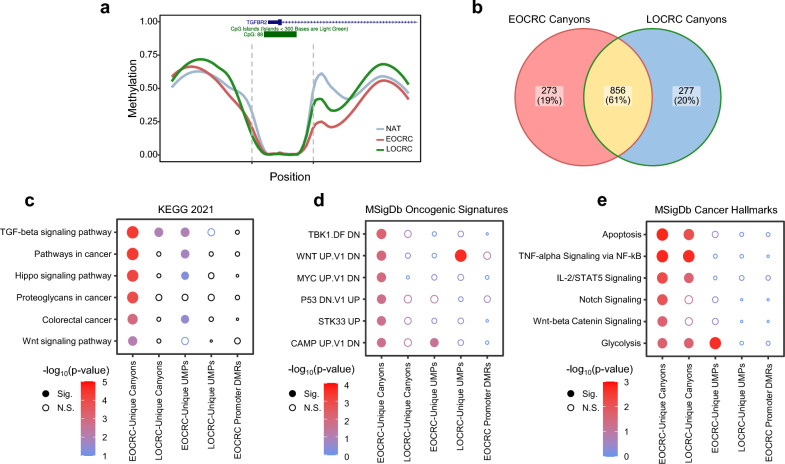
Methylation canyons overlap oncogenic pathway genes and are altered in colorectal cancer. Methylation canyons were defined as undermethylated regions with < 0.1 average methylation values and > 3.5 kb in length. **a** Smoothed line plot of methylation over the *TGFBR2* canyon showing erosion of the downstream canyon border in EOCRC and LOCRC. Dashed lines represent approximate canyon borders. Gene transcript and CpG islands are indicated above; solid blue blocks represent the promoter and transcription start site, arrows represent the gene body and direction of transcription, and green blocks represent CpG islands. **b** Venn diagram of shared and unique gene canyon targets discovered in EOCRC and LOCRC. **c** KEGG pathway enrichment plot for selected top pathways comparing canyons discovered in EOCRC and LOCRC tissues. Undermethylated promoters (< 0.1 methylation) and promoter DMRs discovered in EOCRC are included as controls. **d** MSigDb oncogenic signatures enrichment plot for selected top pathways comparing canyons discovered in EOCRC and LOCRC tissues. **e** MSigDb cancer hallmarks enrichment plot for selected top pathways comparing canyons discovered in EOCRC and LOCRC tissues. CpG, cytosine-guanine dinucleotide; DMR, differentially methylated regions; EOCRC, early-onset colorectal cancer; LOCRC, late-onset colorectal cancer; N.S., not significant; Sig., significant; *TGFBR2*, transforming growth factor-β receptor type 2; UMP, undermethylated promoter

Next, because methylation canyons are linked to transcriptional dysregulation and oncogene expression in cancer, we performed de novo discovery of undermethylated regions (UMRs) using a previously developed Python script [28]. Adjacent UMRs within 500 bp were merged if the resulting region had a mean methylation value ≤ 0.1. We defined methylation canyons as UMRs with a methylation value < 0.1 and length ≥ 3.5 kb. To compare differences in methylation canyons between EOCRC and LOCRC, we included all currently available WGBS datasets of LOCRC tumors from TCGA (*n* = 5). Canyons were characterized by long valleys of low methylation, bordered by sharp plateaus of high methylation. As an example, one methylation canyon overlapped the promoter and body of the transforming growth factor beta receptor type 2 (*TGFBR2*) gene (Fig. 3a). Compared to NATs, we found that EOCRCs and, to a lesser degree, LOCRCs exhibited severe erosion of the downstream canyon border, suggesting increased transcriptional accessibility of *TGFBR2.*

273 EOCRC-unique and 277 LOCRC-unique canyons were identified (Fig. 3b). To identify key pathways, we performed gene set overrepresentation analysis using three databases: KEGG (Fig. 3c), MSigDB Oncogenic Signatures (Fig. 3d), and MsigDB Cancer Hallmarks (Fig. 3e). As a control for non-canyon regions, we compared canyons to undermethylated promoters (UMPs, methylation < 0.1) unique to EOCRC (*n* = 1361) or LOCRC (*n* = 206) and to promoter DMRs detected in the EOCRC vs. NAT analysis (*n* = 1073). EOCRC-unique canyons exhibited strong enrichment in cancer-related pathways, notably the Wnt and TGF-β signaling pathways. Comparatively, LOCRC-unique canyons were significantly enriched only in TGF-β-related pathways. Although the biological and regulatory roles of these canyons require further exploration, these findings mirror previous reports that canyons are involved in oncogene regulation in solid tumors [17]. Interestingly, enrichment was mostly specific to EOCRC, despite approximately equal numbers of EOCRC-unique and LOCRC-unique canyons (Fig. 3b), suggesting that aberrant canyon methylation may be more associated with EOCRC rather than LOCRC tumorigenesis in these cohorts. Weak enrichment in the KEGG gene set occurred with EOCRC-unique UMPs, reflecting some overlap of promoters with canyons (Fig. 3c). However, promoter DMRs were not enriched in any of the cancer-related pathways enriched for in methylation canyons. Although case–control promoter DMR analysis is commonly used in DNA methylation cancer research, our findings show that DMR analysis alone is insufficient to capture gene set enrichment in biologically relevant pathways. This highlights the power of WGBS, which revealed biological insights undetected via traditional promoter DMR analysis.

### Molecular basis of racial disparities between two cohorts of EOCRC patients

**Fig. 4:**
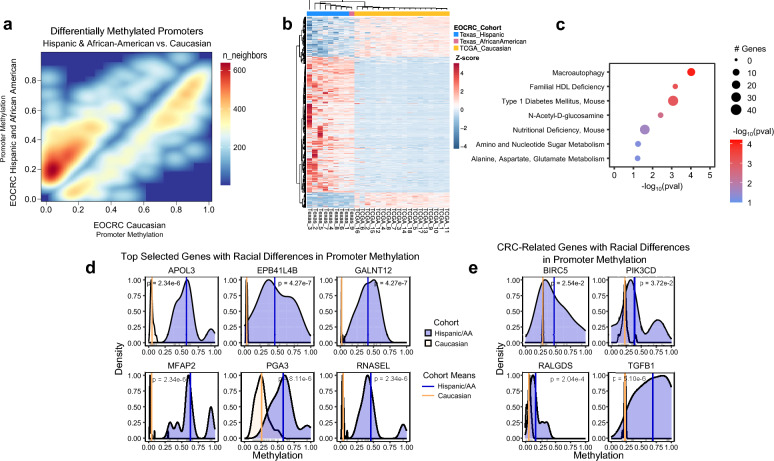
Racial differences in promoter methylation between Caucasian and Hispanic/African American cohorts suggest a molecular basis for EOCRC disparity. Comparison between Hispanic and African American patients (Texas cohort) and Caucasian EOCRC patients (TCGA cohort). **a** Smoothed scatter density plot of 1,168 promoters differentially methylated in Hispanic and African American versus Caucasian EOCRC tissues. Color represents point density (blue, low; red, high). **b** Heatmap of significant differentially methylated promoters, normalized by row-wise z-score. Column annotations denote race. All patients had microsatellite-stable (mismatch repair-proficient) and nonhypermutated tumors. **c** Selected top enriched pathways relating to metabolism from all differentially methylated promoters. **d** Statistical analysis by interquartile range and Wilcoxon ranked sum test identifying genes with significant methylation differences between Hispanic/African American EOCRC tumors and Caucasian EOCRC tumors. Vertical lines indicate mean methylation values of each promoter in the Hispanic/African American (blue) or Caucasian (yellow) cohorts; distributions represent methylation values of all EOCRC tumors. **e** Same as **d** except for colorectal cancer-related genes. AA, African American; CRC, colorectal cancer; EOCRC, early-onset colorectal cancer; HDL, high-density lipoprotein; TCGA, The Cancer Genome Atlas

We investigated differences between EOCRC patients of different racial/ethnic backgrounds by comparing two cohorts: Texas cohort, Hispanic (*n* = 8) or African American (*n* = 1); and TCGA cohort, Caucasian (*n* = 18). The TCGA cohort was selected based on the same inclusion criteria as the Texas cohort. To compare WGBS data (Texas cohort) to HM450K microarray data (TCGA cohort), we first reduced the whole-genome data by subsampling to only CpGs covered in the microarray. Although WGBS and HM450K data are well correlated in comparisons from the same sample [29], we addressed potential batch effects by performing quantile normalization. Contrast score distribution after normalization (measured by the false discovery rate-control tool Clipper [30]) was nearly symmetric around 0.0, suggesting removal of the batch effect (Supplementary Fig. S2). For both cohorts, we then assigned CpGs/probes to a gene promoter based on its location within the 1500-bp promoter window around the TSS. Normalized methylation values for each promoter were calculated by averaging all CpGs assigned to that promoter. pDMRs were defined as those with an absolute methylation difference ≥ 0.1 between cohorts. We then filtered for pDMRs with methylation values in tumors from racial and ethnic minority patients outside the 1st or 3rd quartiles of methylation values in Caucasian tissues (Methods) and used the nonparametric Wilcoxon ranked sum test to detect significant differences between cohorts (*p* < 0.05). We discovered 1168 remaining significant pDMRs between minority and Caucasian patients (Fig. 4a). Unsupervised hierarchical clustering of pDMRs showed race-specific patterns of DNA methylation, clustering cohorts separately from each other (Fig. 4b).

Because diet and metabolism are implicated in dysregulation of DNA methylation and are risk factors for CRC, we then performed gene set overrepresentation analysis to assess enrichment of race-associated pDMRs in metabolism-related pathways. Genes differentially methylated in minority EOCRC patients were significantly enriched in several metabolic pathways, including macroautophagy, a mechanism of metabolic homeostasis modulated by nutrient bioavailability (Fig. 4c). Macroautophagy is reprogrammed by tumor cells in response to increased metabolic demands and is also epigenetically dysregulated by diet [31, 32]. Enrichment in familial high-density lipoprotein deficiency (FHD) and type 1 diabetes pathways suggests a potential genetic component of aberrant methylation in metabolic pathways. Indeed, FHD is caused by apolipoprotein deficiency [33], and the apolipoprotein-encoding gene *APOL3* was one of the top hypermethylated genes in minority, but not Caucasian, EOCRC patients (Fig. 4d). Other enriched pathways included amino acid and nucleotide metabolism gene sets, further supporting a nutrition-related epigenetic component in our minority cohort.

To further assess whether race/ethnicity-associated genes included canonical genes involved in CRC oncogenesis, we overlapped our pDMRs with the Human KEGG 2021 “Colorectal Cancer” pathway database and plotted the top CRC-related genes with racial/ethnic differences (Fig. 4e). Only four CRC-related genes (*BIRC5, PIK3CD, RALGDS,* and *TGFB1*) showed statistically significant methylation differences between racial groups, and the differences were substantially less than the differences noted for the previously identified top six genes (*APOL3*, *MFAP2*, etc.), except for *TGFB1*. This may suggest that in our racial/ethnic minority cohort, EOCRC disparities may be related more to auxiliary risk factors, such as metabolism, diet, and immune function, than to canonical CRC pathways. Together, our findings provide early insights for understanding the epigenetic basis for EOCRC racial disparities and characterize race/ethnicity-specific DNA methylation in our cohort.

## Discussion

This paper describes the first whole-genome methylation study at base-pair resolution of EOCRC in patients from underrepresented populations. We provide to the scientific community high quality, complete methylomes for 9 EOCRC tumors from Hispanic and African American patients. We found that our EOCRC methylomes are distinct from a LOCRC cohort, as methylation canyons in EOCRC but not LOCRC preferentially occurred over genes in cancer-related pathways. Our comparative studies revealed enrichment of metabolism- and cancer-related genes differentially methylated in minority EOCRC patients, and our findings add complementary epigenetic information to a recent report by Holowatyj et al. profiling somatic mutation frequency differences in EOCRC patients from different racial groups [34].

We employed several measures to optimize sample purity and proper representation of sporadic EOCRC in our cohort. Patients with high-microsatellite instability tumors and genetic predispositions to CRC (e.g., Lynch Syndrome, inflammatory bowel disease) were excluded. Because EOCRC tends to arise from the left-sided colon, we also restricted our analysis to left-sided tumors. Tissue blocks were microdissected for tumor tissue and NAT and independently verified by a pathologist. We also assessed whether the tumors were hypermethylated at six traditional CpG island methylator phenotype (CIMP) markers (*RUNX3, IGF2, SOCS1, CACNA1G, MLH1,* and *NEUROG1*) because previous reports indicated that EOCRC tumors are less likely to be CIMP-high [22, 24, 35]. We found that methylation differences between NAT and EOCRC were not significant for any CIMP markers (Supplementary Fig. S3). Therefore, our dataset is widely useful, as existing datasets suffer from a lack of clear markers (except chronological age) differentiating EOCRC from LOCRC.

Our study also adds to growing evidence that methylation canyons likely play a regulatory role in solid tumors, in addition to their known existence in hematopoietic cells. Of note, these methylation canyons differ in size and function from previously described large hypomethylated blocks discovered in solid tumors, which were orders of magnitude larger than canyons (5 kb − 10 Mb; median, ~ 40 kb) and speculated to be involved in heterochromatin structure disruption [36, 37]. While some EOCRC canyons spanned larger regions up to 30 kb, most were < 8 kb (Supplementary Fig. S4) and overlapped genes involved in coherent biological processes.

Importantly, methylation canyons in EOCRC overlapped with genes overrepresented in cancer-related pathways, including the TGF-β and Wnt signaling networks, and aberrant canyon methylation was more prevalent in the EOCRC cohort than in the LOCRC cohort. Previous reports have implicated overactive Wnt signaling and enrichment of differentially expressed genes in focal adhesion and EMT pathways in EOCRC [35, 38]. More active EMT in EOCRC may contribute to its rapid progression and poor prognosis. We discovered that both tumor types also shared an eroded downstream canyon border at *TGFBR2*, which in EOCRC was more pronounced. TGFBR2 is the cognate receptor for TGF-β, a well-established inducer of EMT in cancers, in the canonical TGF-β/SMAD signaling pathway [39]. Further studies, however, are needed to establish the regulatory role of these EOCRC-related canyons. We provide early characterization of the landscape of oncogene-associated methylation canyons in our cohort of 9 EOCRC tumors and highlight similarities and differences between EOCRC and LOCRC.

Most notably, we identified several cancer-related metabolic genes with differential methylation between races in patients with EOCRC (Fig. 4d). As mentioned earlier, *APOL3* is functionally connected to lipid cholesterol metabolism. Recently, APOL3 was identified as the top protein in a screening assay for ferroptosis-related CD8 + T cell infiltration in mismatch repair-proficient CRC, implicating it as an antitumor immunity-promoting tumor suppressor [40]. Other genes include *GALNT12* which encodes for an enzyme involved in O-linked glycosylation, and *RNASEL*, a ribonuclease involved in interferon signaling, which have both been implicated as cancer risk genes in population studies [41, 42]. Of particular interest, *MFAP2* was the top-ranked gene by methylation differences; it was highly methylated in minority patients but unmethylated in most Caucasian patients. MFAP2 is a protein component of extracellular matrix microfibrils and critically controls growth factor signal transduction [43]. In humans, genome-wide association studies (GWAS) have linked the *MFAP2* locus to obesity and type 2 diabetes [44]. In mice, *Mfap2* deficiency is a well-known model of metabolic disease with consistent effects on increased adiposity, insulin resistance, hyperglycemia, and predisposition to diabetes [45, 46]. Considering the strong association between metabolic dysregulation and CRC risk, and the higher rates of obesity and metabolic syndrome in Hispanics and African Americans, we postulate that aberrant DNA hypermethylation at the *MFAP2* promoter may suppress *MFAP2* expression and predispose these minorities to EOCRC or EOCRC risk factors. Clearly, future studies will be needed to elucidate the precise roles of the identified genes in human EOCRC. If confirmed, they could serve as valuable biomarkers for EOCRC risk in racial/ethnic minorities.

We acknowledge several limitations of this study. At the time of our manuscript submission, we had thoroughly searched all publicly available WGBS data on CRC and were able to find only five WGBS-sequenced LOCRC samples. In the future, we hope to obtain additional WGBS datasets to expand the scope of this report and confirm our results. Furthermore, although promoter DNA methylation and gene expression are strongly correlated, we did not verify transcription by RT-PCR or RNA-seq. In the absence of expression data for WGBS samples, we instead evaluated correlations between DNA methylation β-values by 450 K array at gene promoters and mRNA expression of those genes in TCGA-EOCRC (*n* = 59) and LOCRC (*n* = 320) samples for which RNA-seq data was also available (Supplementary Figure S5). Although this is a limited analysis, we confirmed that a majority (65.2%) of genes from the differential methylation study showed a negative association between methylation and mRNA, as expected. This proportion agrees approximately with a previous study evaluating the methylation–expression correlation in TCGA samples across 33 cancer types [47] and reflects the complex relationship between increased DNA methylation and gene repression. For *MFAP2*, a top gene related to EOCRC in underrepresented populations, a weak negative correlation was confirmed (Supplementary Figure S5b). Nonetheless, multi-omic analyses, larger sample sizes, and prospectively collected tumor samples are warranted in future studies. In the analysis of methylation between patients from different racial/ethnic backgrounds, there is only a single EOCRC tumor from a patient of African American descent, severely limiting the ability to draw conclusions regarding the role of DNA methylation in African American EOCRC. We show that this sample shows a high degree of similarity to Hispanic samples in PCA space (Supplementary Figure S6) and are more correlated with Hispanic than Caucasian tumors (Supplementary Figure S7), supporting inclusion into the minority population cohort. However, it is important to recognize the restriction of this limited sample size; ultimately, we include this sample as an important resource for an ethnic background traditionally underrepresented in omic studies. Additionally, lack of sufficient clinical annotation (e.g., treatment response) precluded the ability to correlate methylation phenotypes to clinical outcomes. Nevertheless, we believe our study represents an important first step in characterizing a poorly understood cancer subtype and provides an important resource for basic and clinical investigations.

## Materials and methods

### Study approval and exclusion criteria

This study was approved by the Institutional Review Board (IRB) at Baylor College of Medicine (H-21543). After obtaining informed consent under the IRB approved protocols (H-18245 and H-14435), archived tissue samples were obtained from the Human Tissue Acquisition and Pathology Core of the Dan L. Duncan Cancer Center. All research was performed in accordance with relevant guidelines and regulations.

### Public data sourcing

*Caucasian EOCRC TCGA Cohort (HM450K data):* For the integrative analysis of EOCRC racial disparities in methylation patterns, we used TCGA-COAD datasets from the public repository (https://gdc.cancer.gov/). Complete clinical information was available for 60 TCGA-EOCRC patients, including age, sex, TNM stage, microsatellite instability (MSI) status, and somatic mutations in selected genes (*APC*, *KRAS*, *BRAF*, and *TP53*). Eighteen of these patients met the same criteria as the Texas WGBS Cohort. The DNA methylation level of individual CpG sites was quantified by Illumina Infinium HumanMethylation450 BeadChip arrays, as previously described [48].

*LOCRC TCGA Cohort (WGBS data):* LOCRC patients with WGBS data (*n* = 5) were selected from the TCGA-COAD and TCGA-READ cohorts. Data were accessed from the Legacy Genomic Data Commons Portal from the National Cancer Institute.

### Whole-genome bisulfite sequencing (WGBS)

We used formalin-fixed, paraffin-embedded (FFPE) samples for WGBS analysis. One hematoxylin and eosin (H&E)-stained slide along with 7 − 10 unstained (7-µm-thick) slides was cut. Areas of tumor and NAT were circled on the H&E slides by pathologists (NZK, WA), and corresponding areas from unstained slides were manually microdissected using a razor blade. Genomic DNA (gDNA) was extracted from microdissected FFPE sections using QIAamp DNA FFPE Tissue kit. We used 500 ng gDNA for library preparation and next-generation sequencing (NGS), as described previously [49]. Paired-end 150-bp NGS was performed with planned 25 × coverage on an Illumina Novaseq 6000 platform.

### Quality control and read alignment

FastQC v0.11.9 (https://www.bioinformatics.babraham.ac.uk/projects/fastqc/) was used for general quality checks of sequencing reads in FASTQ files. TrimGalore v0.6.6 (https://github.com/FelixKrueger/TrimGalore) was used in the “paired-end” mode to trim 5’ and 3’ read ends and remove adapter sequences. BSMAP v2.90 (https://code.google.com/archive/p/bsmap/) was used in the “paired-end” mode to align raw FASTQ reads to the hg38 reference genome, which was downloaded from the UCSC Genome Browser (https://hgdownload.soe.ucsc.edu/downloads.html). Samtools v1.11 (https://samtools.sourceforge.net/) was used to remove unmapped reads and sort aligned reads.

### Methylation quantification

Methylation was quantified at the single-CpG level using a Python script adapted from BSMAP, with a minimum of 4 reads covering each CpG (-m 4) in the -x CG mode for CpG methylation (with the -p, -u, -r, and -g options for quality control of reads). For promoter-level methylation analysis, gene promoter regions were defined as 1 kb upstream of the transcription start site (TSS) to 500 bp downstream of the TSS, constituting a 1.5-kb window around the TSS. A promoter browser extensible data file was obtained from the UCSC Genome Browser Table Browser (http://genome.ucsc.edu/cgi-bin/hgTables) and used to average methylation across pre-defined regions using the BedRatio command of CAMDA.py (https://github.com/JiejunShi/CAMDA).

### Global methylation and methylation canyon analyses

To compare global methylation, the genome was split into 2-kb nonoverlapping sliding windows using the “makewindows” function of Bedtools v2.29.2. Genomic regions on the X chromosome were removed from all subsequent analyses to account for DNA methylation-mediated X chromosome inactivation in female patients. Methylation values were then averaged over each sliding window using the Bedtools “map” function. To discover methylation canyons, undermethylated regions (UMRs) were first identified for individual samples using a Python script, which solves a beta-binomial on raw sequencing counts using a Hidden Markov Model [28]. Adjacent UMRs were merged using the MergeUMR.py script of the PopCanyon tool (https://github.com/JiejunShi/PopCanyon) if they occurred within 500 bp of each other and the resulting merged UMR retained an average methylation value < 0.1. UMRs > 3.5 kb were designated as canyons. Canyons were annotated to a gene if the canyon overlapped any part of a gene using the “intersect” tool of Bedtools v2.29.2. Canyons discovered in each sample were then merged, and canyons found in < 50% of samples within each group (NAT, EOCRC, LOCRC) were removed. Methylation canyons were visualized with the smooth function of the ggplot2 R package using the locally estimated scatterplot smoothing method and a span of 0.2. Smoothed line plots of methylation canyons were annotated with gene diagrams exported from the UCSC Genome Browser (https://genome.ucsc.edu/cgi-bin/hgTracks).

### Differential methylation analysis

Metilene v0.2–8 (https://www.bioinf.uni-leipzig.de/Software/metilene/) was used for all differential methylation analyses. First, single-CpG methylation quantified files were converted to bedGraph format using a custom R script. Bedtools v2.29.2 (https://bedtools.readthedocs.io/) was then used to merge samples, and the resulting merged file was used as input for Metilene. For the unbiased differentially methylated CpG position (DMP) analysis, individual CpGs were statistically tested for differential methylation using Metilene in the de novo DMP mode (-f 3). For the unbiased differentially methylated region (DMR) analysis, Metilene was used in the de novo DMR identification mode (-f 1) with default parameters. For differential methylation at pre-defined promoter regions, Metilene was used in the pre-defined regions mode (-f 2) with default parameters and the previously described promoter region bedfile. Individual CpGs and DMRs were annotated according to their genomic or CpG features using the annotatr R package. All differentially methylated promoter regions are provided in Supplementary File 1 as a tab-separated file.

### Gene set overrepresentation analysis

*Metabolism-related gene sets:* Race-associated pDMRs were used as the input gene list for enrichment analysis using the EnrichR R package. Metabolism-related terms were selected from the KEGG 2021 Human, Gene Ontology Biological Process, DisGeNet (https://www.disgenet.org/), Human Metabolome Database Metabolites (https://hmdb.ca/), and GEO perturbation databases. Enrichment was performed on these metabolism-related terms and plotted using ggplot2.

*Methylation canyon gene set overrepresentation:* EnrichR was used to perform gene set overrepresentation analysis with all genes annotated to unique canyons discovered in the EOCRC or LOCRC cohorts as input. KEGG 2021 Human, MSigDB Oncogenic Signatures, and MSigDB Cancer Hallmarks databases were queried and enrichment p-values were plotted using ggplot2 for EOCRC-unique canyons, LOCRC-unique canyons, EOCRC-unique undermethylated promoters (UMPs), LOCRC-unique UMPs, and promoter DMRs discovered in the EOCRC vs. NAT differential methylation analysis. A unique UMP was defined as a promoter with a methylation value < 0.1 in one type of CRC but not in the other type of CRC.

### Identification of race-associated differentially methylated promoter regions

The Caucasian EOCRC cohort included 18 patients from the TCGA-COAD dataset who were diagnosed at < 50 years of age, had tumors annotated as MMR-proficient and nonhypermutator, and with their race annotated as Caucasian. WGBS methylation values from the Texas cohort were overlapped using a custom R script to keep only CpGs covered by probes in the Illumina HumanMethylation 450 K microarray. WGBS and HM450K methylation values were then quantile normalized, and their contrast score distributions were assessed for batch effect using Clipper [30]. Normalized methylation values were then assigned to genes based on their location within each 1.5-kb promoter region using the “intersect” function of Bedtools v2.29.2, and all methylation values for each promoter were averaged. Promoters were designated as differentially methylated (promoter DMRs [pDMRs]) if the absolute methylation difference between Hispanics/African American (Texas cohort) and Caucasians (TCGA cohort) was > 0.1. pDMRs were further filtered by interquartile range (IQR). The 1st quartile (Q1) and 3rd quartile (Q3) of pDMR values in the Caucasian cohort were calculated using the “quantile” function of base R for each promoter. pDMRs were retained if their average promoter methylation value across Hispanic/African American tumors was < Q1 (to – 1.5 × IQR) or > Q3 (to + 1.5 × IQR) of the Caucasian promoter values. Wilcoxon ranked sum test was used to test for significance, with a false discovery rate-adjusted threshold of p < 0.05. Significant pDMRs were visualized using the “smoothScatter” base R function, and heatmaps were drawn using the “aheatmap” function of the NMF v0.17.6 R package (https://nmf.r-forge.r-project.org/index.html). Density plots showing the distribution of promoter methylation values in each cohort were plotted using the ggplot2 R package.

## Conclusions

Sporadic colorectal cancers in patients under the age of 50 are rapidly increasing in incidence globally, presenting an important health crisis. Despite this trend, EOCRC remains understudied. Its molecular mechanisms, both unique and shared with LOCRC, are unsolved and poorly characterized. Our contribution of base-pair resolution whole-methylome profiles represents a crucial step forward in EOCRC research. While larger-scale EOCRC datasets remain limited by cost and cohort recruitment, our findings provide preliminary evidence that the epigenome of EOCRC is distinct from that of LOCRC. We also find that biologically coherent metabolic and EMT pathways are epigenetically dysregulated in EOCRC patients from underrepresented minority ancestries, suggesting a role for DNA methylation in the disparity in cancer incidence and mortality among these patients.

## Supplementary Information

Additional file 1.

Additional file 2 .

## Data Availability

The WGBS data in this study have been deposited in NCBI’s Gene Expression Omnibus and are accessible through GEO Series accession number GSE284325.
