# Supplementary material for: DNA methylation profiling at base-pair resolution reveals unique epigenetic features of early-onset colorectal cancer in underrepresented populations
Source: Clin Epigenetics. 2025 Jan 22;17:11. doi: 10.1186/s13148-025-01817-z (PMC11753045; doi:10.1186/s13148-025-01817-z)
Supplement: Supplementary file 1 — Additional file 1. [file 13148_2025_1817_MOESM1_ESM.pdf]

Fig. S1.

a

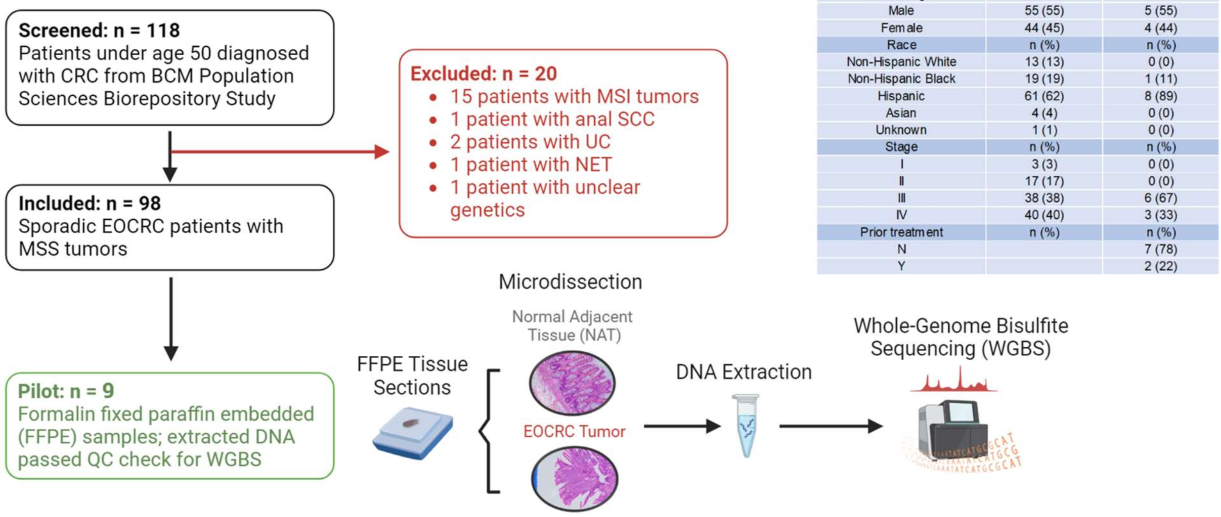

b

	Texas Cohort	WGBS Samples
	n = 98	n = 9
Age Range	21-49	34-48
Median age	43	45
Male	55 (55)	5 (55)
Female	44 (45)	4 (44)
Race	n (%)	n (%)
Non-Hispanic White	13 (13)	0 (0)
Non-Hispanic Black	19 (19)	1 (11)
Hispanic	61 (62)	8 (89)
Asian	4 (4)	0 (0)
Unknown	1 (1)	0 (0)
Stage	n (%)	n (%)
I	3 (3)	0 (0)
II	17 (17)	0 (0)
III	38 (38)	6 (67)
IV	40 (40)	3 (33)
Prior treatment	n (%)	n (%)
N		7 (78)
Y		2 (22)

EOCRC patient selection and cohort demographics. a) Flow chart showing patient selection and excluded patients for the Texas cohort, as well as the subsequent analysis. **b)** Patient demographics and characteristics of the full Texas cohort (n=98) and the WGBS samples (n=9). BCM, Baylor College of Medicine; CRC, colorectal cancer; EOCRC, early-onset colorectal cancer; MSI, microsatellite instability; MSS, microsatellite stable; NET, neuroendocrine tumor; QC, quality control; SCC, squamous cell carcinoma; UC, ulcerative colitis.

Fig. S2.

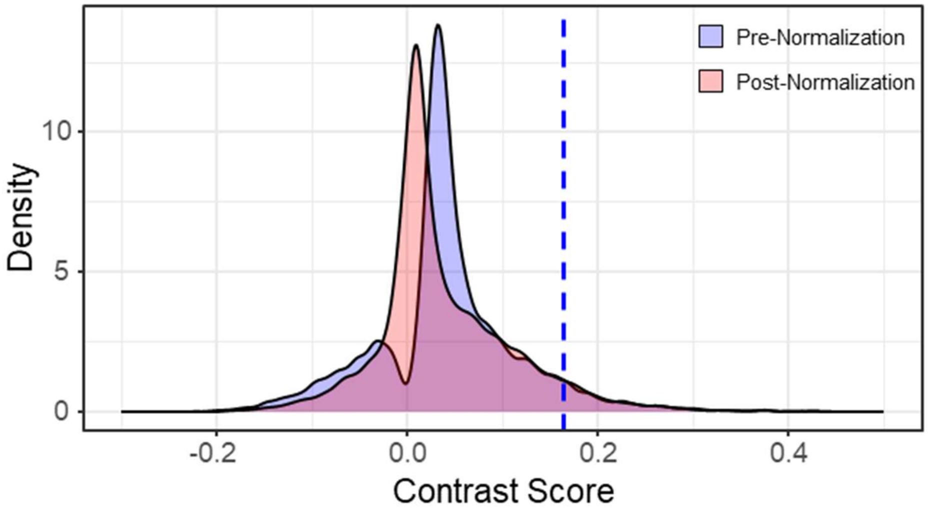

Quantile normalization removes batch effects between WGBS and HM450K data. The WGBS data (Texas cohort) were subset to HM450K probe locations, then quantile normalized with HM450K data (The Cancer Genome Atlas cohort). Density curves of Clipper-generated contrast scores are plotted. The blue curve represents pre-normalization data, and the red curve represents post-normalization data. Following normalization, contrast scores are more symmetric around a 0.0 contrast score. The dashed blue line indicates the false discovery rate-control threshold for differentially methylated genes. WGBS, whole-genome bisulfite sequencing.

Fig. S3.

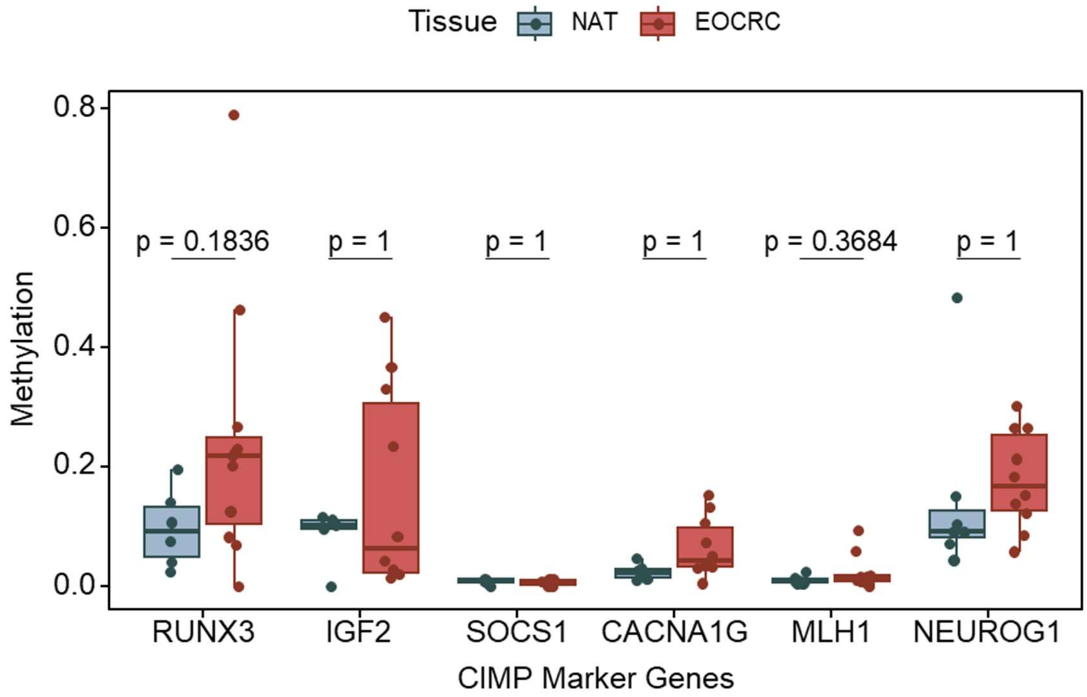

Promoter methylation at CpG-island methylator phenotype (CIMP) genes. Methylation at promoters at common CIMP marker genes is shown as boxplots for normal adjacent tissue and EOCRC tissue. Dots indicate CIMP gene methylation of single samples. Adjusted p-values shown for each gene were determined using Student's t-test with Bonferroni correction. EOCRC, early-onset colorectal cancer; NAT, normal adjacent tissue.

Fig. S4.

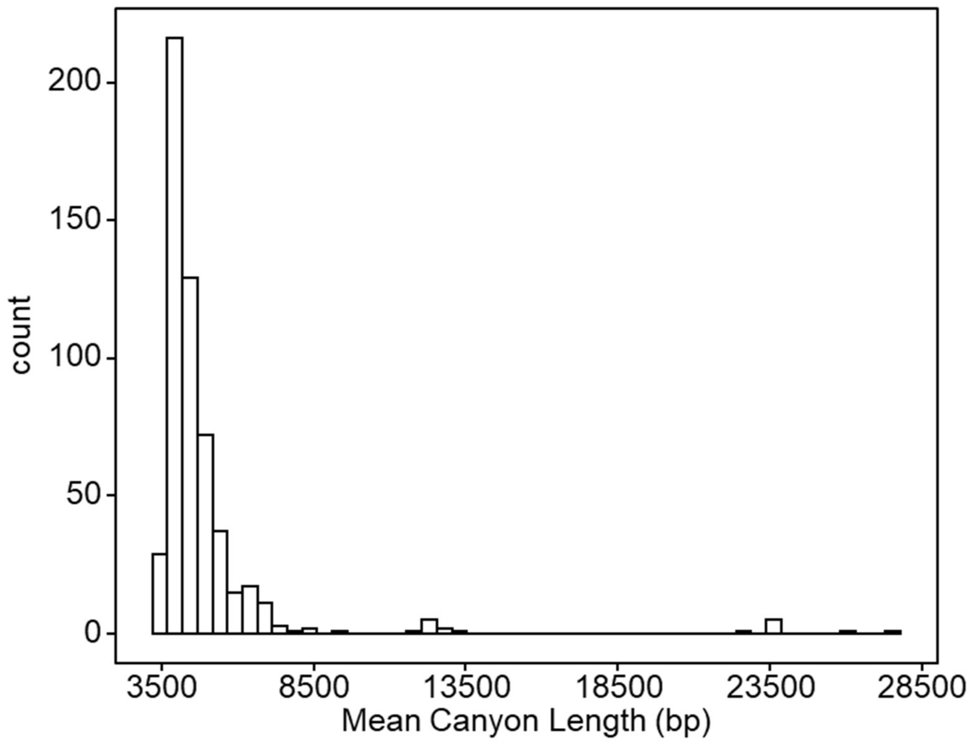

Distribution of methylation canyon length. Histogram of the distribution of methylation canyon lengths in all early-onset colorectal cancer and late-onset colorectal cancer canyons. Canyons are defined as regions that are undermethylated (methylation <0.1) and >3500 bp in length.

Fig. S5.

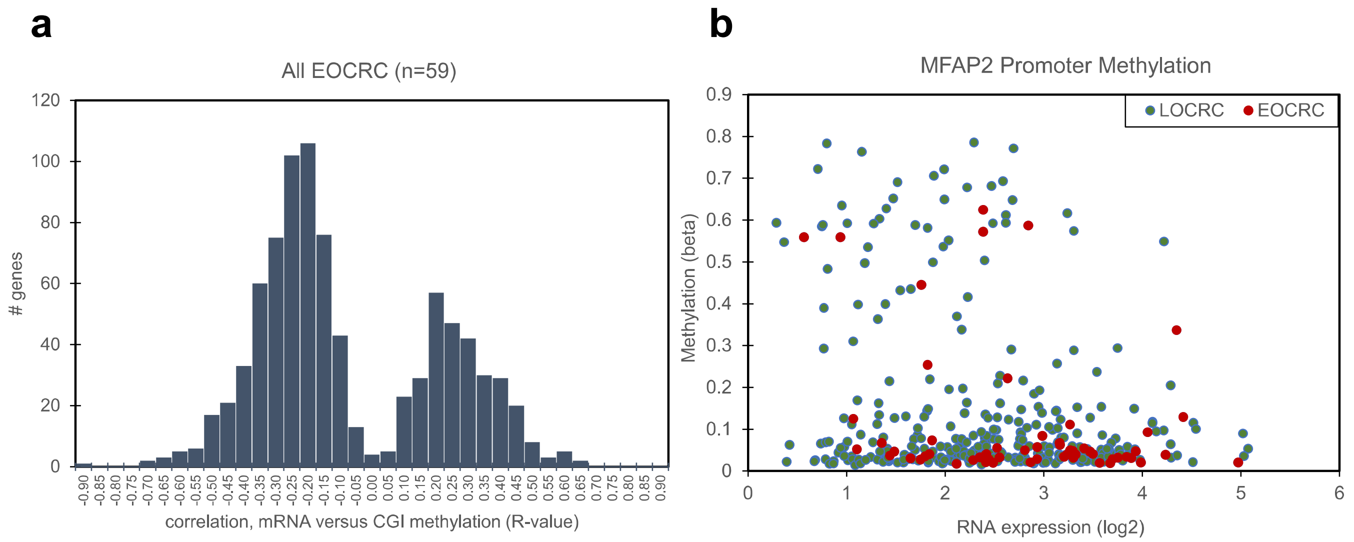

Correlation between promoter DNA methylation and mRNA expression in CRC patients from the TCGA cohort. (a) Histogram of directional correlation (*R*) between mRNA expression and promoter CGI methylation at 1,168 genes for the full TCGA EOCRC cohort (n = 59). **(b)** Scatterplot of *MFAP2* mRNA expression vs. methylation β-value in EOCRC (n = 59) and LOCRC (n = 320) samples from TCGA.

Fig. S6.

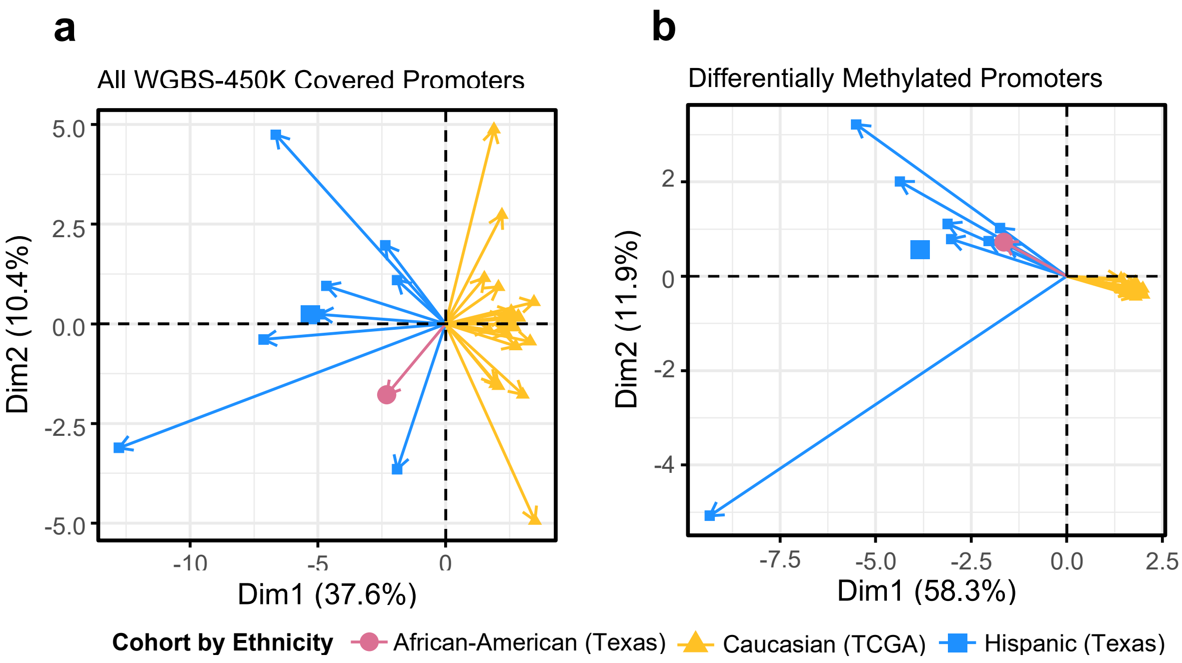

Principal component analysis (PCA) of methylation between Texas and TCGA cohorts. (a) PCA on methylation values averaged over all gene promoter regions covered by both WGBS (Texas cohort) and 450K microarray (TCGA cohort) samples. Samples are labeled by their ethnicity and cohort. **(b)** Same as (a) but restricted to gene promoter regions differentially methylated between the Texas and TCGA cohorts.

Fig. S7.

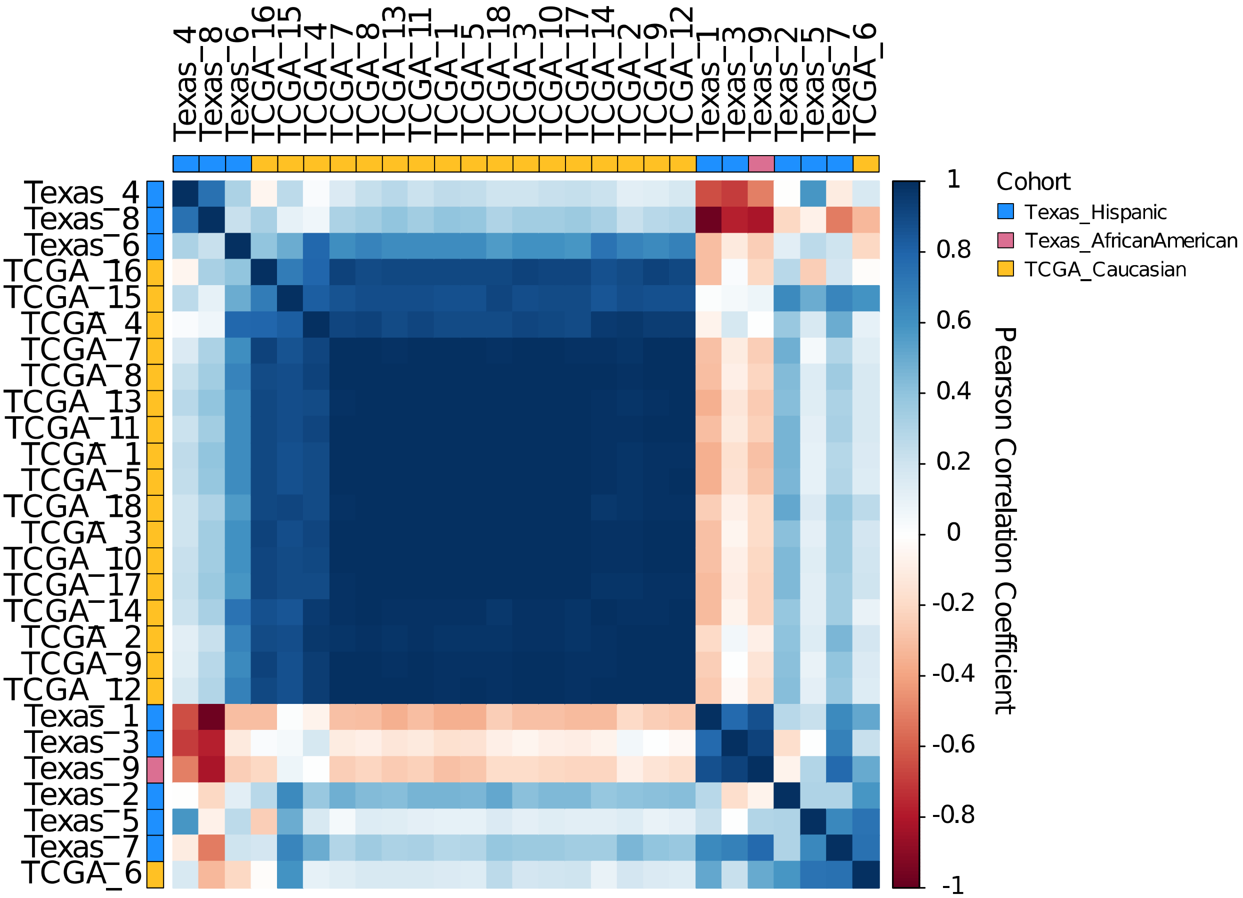

Correlation matrix of methylation profiles from EOCRC tumors in the Texas and TCGA cohorts. Methylation values at the top 6 selected gene promoters differentially methylated between cohorts were used to calculate pairwise Pearson correlation coefficients, visualized as a matrix. Ethnicity and cohort of each sample is provided as column and row annotations. Sample order was determined by hierarchical clustering.

Table S1.

ID	Race	Sex	Age	Stage	Histology	Treatment Prior to Resection
N1	Hispanic	M	44	III	Normal Adjacent Tissue	Total Neoadjuvant Treatment
N2	Hispanic	F	44	IV	Normal Adjacent Rectosigmoid Tissue	Total Neoadjuvant Treatment
N3	Hispanic	M	47	III	Normal Adjacent Sigmoid Tissue	No Treatment Before Surgery
N4	Hispanic	F	48	III	Normal Adjacent Sigmoid Tissue	No Treatment Before Surgery
N5	Hispanic	F	45	III	Normal Adjacent Sigmoid Tissue	No Treatment Before Surgery
N6	Hispanic	M	46	IV	Normal Adjacent Rectal Tissue	No Treatment Before Surgery
N7	Black	M	45	III	Normal Adjacent Tissue	No Treatment Before Surgery
T1	Hispanic	M	44	III	Moderately Differentiated Rectal Adenocarcinoma	Total Neoadjuvant Treatment
T2	Hispanic	F	44	IV	Moderately Differentiated Rectosigmoid Adenocarcinoma	Total Neoadjuvant Treatment
T3	Hispanic	M	47	III	Moderately Differentiated Sigmoid Colon Cancer	No Treatment Before Surgery
T4	Hispanic	F	48	III	Moderately Differentiated Sigmoid Colon Cancer	No Treatment Before Surgery
T5	Hispanic	F	45	III	Moderately Differentiated Sigmoid Colon Cancer	No Treatment Before Surgery
T6	Hispanic	M	46	IV	Rectal Adenocarcinoma Arising from Tubulovillous Adenoma with High Grade Dysplasia	No Treatment Before Surgery
T7	Hispanic	F	43	III	Moderately Differentiated Sigmoid Colon Cancer	No Treatment Before Surgery
T8	Hispanic	M	48	III	Moderately Differentiated Sigmoid Colon Cancer	No Treatment Before Surgery
T9	Black	M	34	IV	Moderately Differentiated Rectosigmoid Adenocarcinoma	No Treatment Before Surgery

Texas cohort clinical characteristics. All patients had early-onset, mismatch repair (MMR)-proficient, nonhypermutated colorectal cancer and no known genetic syndrome predisposing to colorectal cancer. F, female; M, male; N1 to N7, normal adjacent tissues 1 to 7; T1 to T9, tumors 1 to 9.
